# Alpha 1 Antitrypsin Gene Therapy Extends the Lifespan of Lupus-Prone Mice

**DOI:** 10.1016/j.omtm.2018.10.007

**Published:** 2018-10-18

**Authors:** Ahmed Samir Elshikha, Ye Yuan, Yuanqing Lu, Mong-Jen Chen, Georges Abboud, Mohammad Ahsanul Akbar, Henrike Plate, Hedwig Wolney, Tanja Hoffmann, Eleni Tagari, Leilani Zeumer, Laurence Morel, Sihong Song

**Affiliations:** 1 Department of Pharmaceutics, College of Pharmacy, University of Florida, Gainesville, FL 32610, USA; 2 Department of Pathology, Immunology and Laboratory Medicine, University of Florida, Gainesville, FL 32610, USA; 3 Department of Pharmaceutics, Zagazig University, Zagazig, Sharkia, Egypt

**Keywords:** alpha 1 antitrypsin, AAT, systemic lupus erythematosus, SLE, rAAV, dendritic cells, DCs, autoimmunity, autoantibodies, life span

## Abstract

Systemic lupus erythematosus (SLE) is a heterogeneous autoimmune disease characterized by high levels of pathogenic autoantibodies and tissue damage. Multiple studies showed that dendritic cell (DC) activation plays a critical role in SLE pathogenesis. Human alpha 1 antitrypsin (hAAT) is a serine proteinase inhibitor with potent anti-inflammatory and cytoprotective properties. In this study, we first examined the effects of hAAT on the functions of DCs from lupus-prone mice, and we showed that hAAT treatment efficiently inhibited CpG- (TLR9 agonist) induced activation of bone marrow-derived conventional and plasmacytoid DCs as well as the production of pro-inflammatory cytokines. The hAAT treatment also attenuated DC help for B cell proliferation and immunoglobulin M (IgM) production. We next tested the protective effect of hAAT protein and gene therapy using recombinant adeno-associated virus 8 (rAAV8-CB-hAAT) in a spontaneous lupus mouse model, and we showed that both treatments decreased autoantibody levels. Importantly, rAAV8-CB-hAAT did not induce an immune response to its transgene product (hAAT), but it showed more pronounced therapeutic effects in reducing urine protein levels and extending the lifespan of these mice. These results indicate that AAT has therapeutic potential in the treatment of SLE in humans.

## Introduction

Systemic lupus erythematosus (SLE) is a life-threatening autoimmune disease for which there is no cure. Most treatment options are symptomatic and nonspecific, using anti-malarial, corticosteroids, and immunosuppressive drugs, which have severe side effects.^1^ Therefore, there is an unmet need for a safe and effective therapy for SLE. Multiple studies have shown that dendritic cells (DCs) play a vital role in SLE pathogenesis.^2^, ^3^, ^4^ DCs are antigen-presenting cells (APCs) that consist of conventional DCs (cDCs) and plasmacytoid DCs (pDCs), both of which are activated by Toll-like receptor (TLR) signaling.^2^, ^5^, ^6^ TLR3, 7/8, and 9 are endosomal receptors that recognize viral double-stranded RNA, viral single-stranded RNA, and viral or bacterial DNA containing unmethylated CpG motifs, respectively.^5^ Stimulation of these TLRs leads to the production of pro-inflammatory cytokines, such as interleukin-6 (IL-6), IL-12, tumor necrosis factor alpha (TNF-α), IL-1β, and type I interferons (IFN-I). These pro-inflammatory cytokines have pleiotropic effects in promoting SLE.^7^, ^8^ Elevated levels of IFN-I correlate with disease activity in lupus patients.^9^

One characteristic feature of SLE is the production of elevated levels of autoantibodies, particularly those recognizing double-stranded DNA, due to aberrant autoreactive B cells.^7^, ^10^ Although T cell help is important for B cell production of autoantibodies,^7^, ^10^ DCs play an important role in B cell development and function by producing B cell-activating factor (BAFF), IL-6, IL-12, and IFN-I.^11^, ^12^ Therefore, targeting DC activation and functions can lead to the inhibition of autoantibody production, which in turn alleviates SLE progression. Targeting DCs could also have an indirect therapeutic effect by decreasing T cell help to autoreactive B cells.

Human alpha-1 antitrypsin (hAAT) is a multifunctional protein. The well-known function of hAAT is to inhibit neutrophil elastase and prevent emphysema.^13^ hAAT also has anti-inflammatory and immunoregulatory properties. Studies have shown the protective effects of hAAT in several disease conditions, including transplant rejection,^14^ ischemia-reperfusion injury,^15^, ^16^ graft-versus-host disease,^17^ stroke,^18^ osteoporosis,^19^, ^20^ rheumatoid arthritis,^21^ and type 1 diabetes.^22^, ^23^, ^24^ Recently, we have shown that hAAT protein therapy ameliorates disease outcomes in MRL-lpr lupus-prone mice.^25^ However, the mechanism underlying the protective effect of hAAT and whether it expands the lifespan of lupus-prone mice remain to be investigated. In addition, hAAT gene therapy for lupus has never been reported.

Recombinant adeno-associated virus (rAAV) vector is a promising vector that can mediate long-term and high levels of transgene expression in a wide variety of tissues. At least 10 serotypes of rAAV vectors have been developed and used in animal models and in humans.^26^, ^27^, ^28^ We have developed an rAAV vector to express sustained and therapeutic levels of hAAT, which has been used in mouse models^29^, ^30^ and tested in humans.^28^ rAAV8-hAAT vector failed to transduce DCs and resulted in immune tolerance to hAAT in the non-obese diabetic (NOD) mouse model of type 1 diabetes, suggesting this vector presents an advantage for immunosuppressive therapy.^31^ Indeed, using this vector, we have shown that hAAT gene therapy delayed arthritis development in the collagen-induced arthritis mouse model, and it prevented bone loss in an osteoporosis mouse model.^19^, ^21^ In this study, we tested the protective effect of hAAT on DCs and hAAT protein and gene therapy on lupus development in a lupus-prone mouse model, the NZM2410 strain and its congenic derivative B6.NZM2410.Sle1.Sle2.Sle3 (B6.TC).^32^

## Results

### hAAT Treatment Inhibits DC Activation Induced by a TLR9 Agonist

We have demonstrated that hAAT inhibited the maturation and function of bone marrow-derived DCs (BMDCs) obtained from non-autoimmune C57BL/6 mice upon stimulation with lipopolysaccharide (LPS) (TLR 4 agonist) or CpG-ODN 1826 (TLR9 agonist).^25^ To further evaluate the effect of hAAT on DCs from lupus mice, we used BMDCs from the B6.TC strain.^33^ BM cDCs from B6.TC mice were induced by IL-4 and granulocyte-macrophage colony-stimulating factor (GM-CSF) in the presence or absence of hAAT. At day 4, cells were stimulated with CpG for an additional 24 hr, and then they were harvested for the evaluation of cDC maturation and cytokine production. We selected CpG, a TLR9 agonist, to activate DCs since the TLR9 pathway plays a pivotal role in lupus pathogenesis.^34^

**Figure 1:**
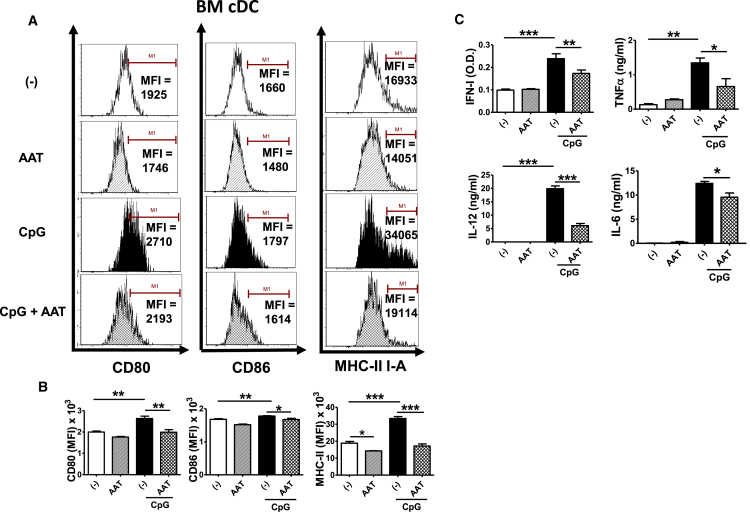
hAAT Inhibited BM cDC Maturation and Expression of Costimulatory Molecules BM cDCs from B6.TC mice were differentiated *in vitro* with or without hAAT for 4 days and then stimulated or not with 10 μg/mL CpG for an additional 24 hr prior to fluorescence-activated cell sorting (FACS) analysis. (A) Representative FACS plots showing the MFI (mean fluorescence intensity) of cDC costimulatory molecules CD80, CD86, and MHC-II. (B) Statistical analysis for CD80, CD86, and MHC-II expression on cDCs. (C) IFN-I, TNF-α, IL-12, and IL-6 levels in supernatants. Data were expressed as the mean ± SEM and analyzed by one-way ANOVA using Tukey’s post hoc test corrections. n = 3; *p < 0.05, **p < 0.01, and ***p < 0.001.

**Figure 2:**
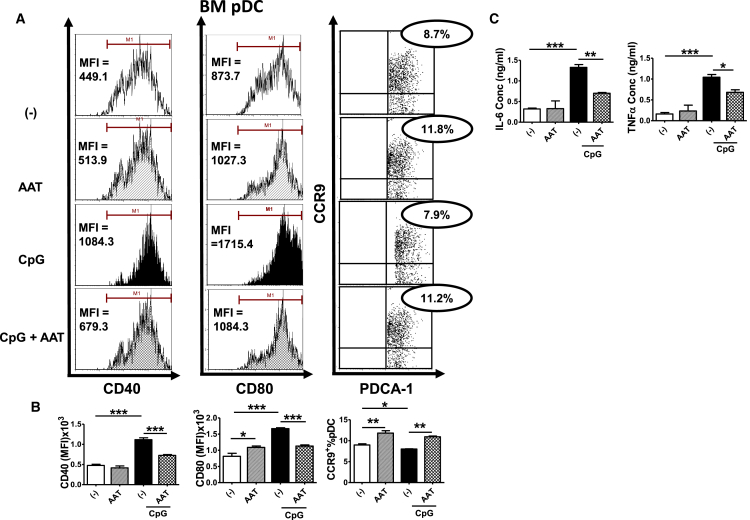
hAAT Inhibited BM pDC Maturation and Cytokine Secretion BM pDCs from B6.TC mice were differentiated *in vitro* using Flt3l with or without hAAT for 8 days and then stimulated or not with 10 μg/mL CpG for an additional 24 hr prior to FACS analysis. (A) Representative FACS plots showing the MFI of costimulatory molecules CD40 and CD80 and the frequency of PDCA-1^+^ CCR9^+^ cells, gated on PDCA-1^hi^ CD11c^int^. (B) Statistical analysis for CD40 and CD80 expression and frequency of the CCR9^+^ PDCA-1^+^ in pDCs. (C) IL-6 and TNF-α levels in supernatants. Data were analyzed by one-way ANOVA using Tukey’s post hoc test corrections. n = 3; *p < 0.05, **p < 0.01, and ***p < 0.001.

The hAAT treatment inhibited CpG-induced expression of the costimulatory molecules CD80, CD86, and MHC-II (Figures 1A and 1B). In addition, AAT treatment markedly attenuated CpG-induced IFN-I, TNF-α, IL-12, and IL-6 production (Figure 1C). Similarly, we tested the effect of hAAT on BM pDCs also obtained from B6.TC mice. hAAT decreased CpG-induced expression of the costimulatory molecules CD40 and CD80 (Figures 2A and 2B). The chemokine receptor CCR9 expressed on pDCs can be downregulated in response to stimulation, and it is commonly used as a marker for tolerogenic DCs.^35^ We found that CpG treatment reduced CCR9 expression while hAAT treatment significantly increased CCR9 expression in B6.TC pDCs (Figures 2A and 2B). We also found that the levels of IL-6 and TNF-α were significantly reduced in hAAT-treated BM pDCs (Figure 2C). These results demonstrated that the AAT treatment significantly inhibited the CpG-induced maturation and expression of costimulatory molecules in cDCs and pDCs from lupus-prone mice.

### hAAT Treatment Attenuated the Capacity of cDCs in Stimulating B Cells

**Figure 3:**
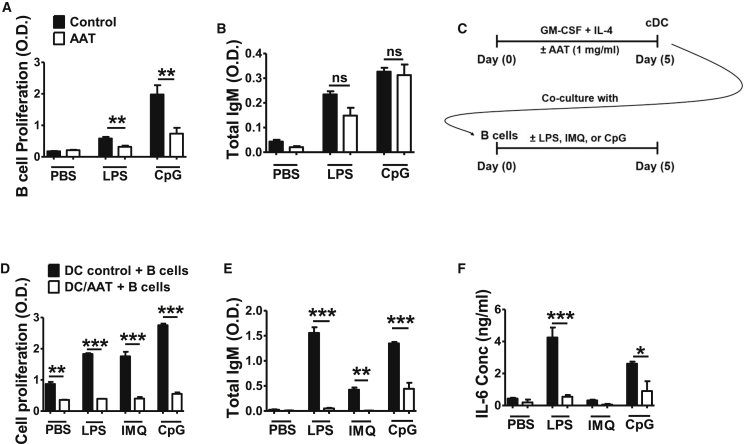
Effect of hAAT-Treated BM cDCs on B Cell Functions (A and B) Splenic B cells from C57BL/6 (B6) mice were purified and cultured *in vitro* with or without AAT in the presence or absence of LPS or CpG for 48 hr. (A) B cell proliferation measured by Cell Titer 96 AQ_ueous_ One Solution Cell Proliferation Assay. (B) Total IgM secretion in supernatants. (C–F) B6 BM cDCs were treated with or without hAAT and co-cultured with B cells. (C) Co-culture experimental design. BM cDCs were differentiated *in vitro* with or without AAT for 5 days. CD11c^+^ cells were purified then co-cultured with purified B cells with or without LPS, IMQ, or CpG for 5 days. (D) Total cell proliferation. (E and F) Total IgM (E) and IL-6 (F) in the co-culture supernatant. Data were expressed as the mean ± SEM and analyzed by one-way ANOVA using Tukey’s post hoc test corrections. n = 3; *p < 0.05, **p < 0.01, and ***p < 0.001; ns, not significant.

Since hAAT inhibits DC activation, we wanted to test whether this inhibition could be extended to their effect on B cells. We first tested the effect of hAAT directly on B cells from normal mice. As shown in Figure 3A, hAAT reduced B cell proliferation induced by LPS or CpG stimulation. However, hAAT treatment did not inhibit LPS- or CpG-induced immunoglobulin M (IgM) production (Figure 3B).

We next tested the effect of hAAT-treated cDCs on B cells in the presence or absence of LPS, Imiquimod (IMQ, a TLR7 agonist), and CpG (Figure 3C). When B cells were co-cultured with untreated DCs, cell proliferation was stimulated by the TLR agonists. However, cell proliferation was significantly lower with hAAT-treated DCs in all conditions, with or without three TLR agonists (Figure 3D). Similarly, with hAAT-treated DCs, the productions of IgM and IL-6 were significantly lower than they were with control DCs (Figures 3E and 3F). These results indicated that hAAT treatment directly inhibits B cell proliferation, but it has no or minimal effect on antibody secretion. More importantly, treatment with hAAT attenuated the ability of cDCs to enhance B cell functions. The indirect effect of hAAT on B cells through DCs appears to be greater than the direct effect on B cells, since a reduction of IgM secretion was observed only in the co-cultures with hAAT-treated DCs (Figure 3B versus Figure 3E).

### hAAT Protein and Gene Therapy Reduced Autoantibody Production in Lupus-Prone Mice

To investigate the effect of hAAT on lupus development, we performed hAAT protein and gene therapy treatment in NZM2410 mice, the parental strain of B6.TC mice that spontaneously develops severe early-onset glomerulonephritis from 20 to 40 weeks of age.^36^ Female NZM2410 mice were treated with PBS as a control, clinical-grade AAT (2 mg every 3 days) as protein therapy, or rAAV8-CB-hAAT vector (1 × 10^11^ vg) as a gene therapy. The treatment started at 8 weeks of age, before the presence of high levels of autoantibodies and clinical disease. At 22 weeks of age, we sacrificed all animals for immunological and pathological examinations.

**Figure 4:**
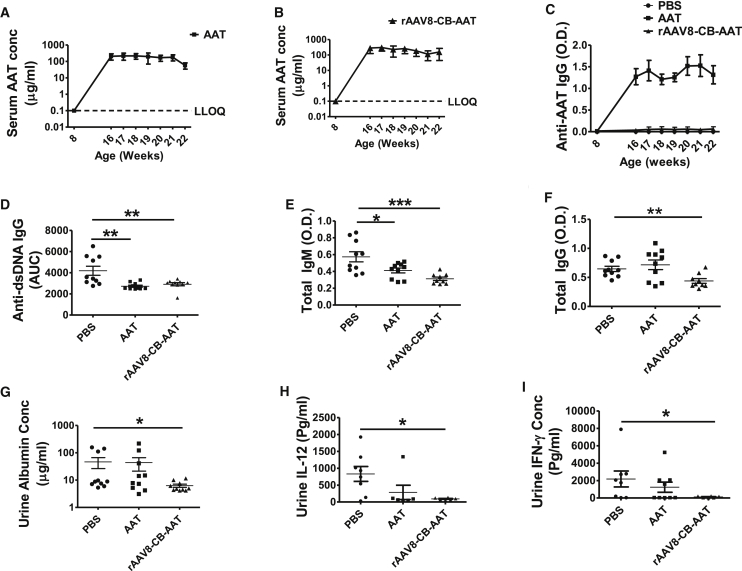
hAAT Inhibited Antibody Production and Proteinuria in NZM2410 Mice 8-week-old female NZM2410 mice (N = 10) were treated with PBS, AAT, or rAAV8-CB-hAAT and sacrificed at 22 weeks of age. (A) Serum hAAT levels in the AAT protein-treated group. The dashed line shows the lower limit of quantification (LLOQ). Results from the PBS-treated group were below LLOQ and are not shown. (B) Serum hAAT levels in the rAAV8-CB-hAAT-treated group. (C) Serum anti-hAAT IgG levels in the hAAT- and rAAV8-CB-hAAT-treated groups. Results from the PBS-treated group were below LLOQ. (D) Serum anti-dsDNA IgG levels at 8 (prior treatments), 16, 17, 18, 19, 20, and 21 weeks of age were detected by ELISA. The area under the curve (AUC) for each animal was calculated, and the average of AUC for each group was plotted and analyzed (E and F) Serum levels of total IgM (E) and IgG (F) at 22 weeks of age. Data were expressed as the mean ± SEM and analyzed by one-way ANOVA using Tukey’s post hoc test corrections. (G–I) Detectable levels of urine albumin (G), IL-12 (H), and IFN-γ (I) levels at 22 weeks of age. PBS versus rAAV-CB-hAAT was expressed as the mean ± SEM and analyzed by two-tailed Mann-Whitney t test. *p < 0.05, **p < 0.01, and ***p < 0.001.

As expected, serum hAAT was detected in the protein therapy group (∼200 μg/mL) (Figure 4A) and the rAAV8-CB-hAAT-treated group (100–300 μg/mL) (Figure 4B). Serum anti-hAAT immunoglobulin G (IgG) was detected in the hAAT protein therapy group, but not in the rAAV8-CB-hAAT-treated group (Figure 4C), as previously observed in the arthritis^21^ and diabetes models.^23^ Importantly, both hAAT protein and gene therapy reduced the serum levels of anti-double-stranded DNA (dsDNA) IgG (Figure 4D), which is a hallmark autoantibody in lupus. Both therapies decreased levels of total IgM (Figure 4E), and rAAV8-CB-hAAT gene therapy decreased total IgG in the circulation (Figure 4F). Furthermore, rAAV8-CB-hAAT gene therapy decreased urine albumin, IL-12, and IFN-γ levels as compared to the control group (Figures 4G–4I), indicating an improvement of kidney function and a reduction of renal inflammation. The protein therapy did not reduce total IgG levels, possibly due to the fact that hAAT protein induces an anti-AAT IgG response in mice (Figure 4F). As expected, neither treatment had an effect on renal pathology histology scores (data not shown), due to very low disease severity at this early time point. These results demonstrated that AAT protein and gene therapy were effective to prevent the production of lupus-related autoantibodies, suggesting the suppression of autoreactive B cells. In addition, the gene therapy showed a renal protective effect.

### Effect of Preventive hAAT Treatment on Lupus DCs and B and T Cells

**Figure 5:**
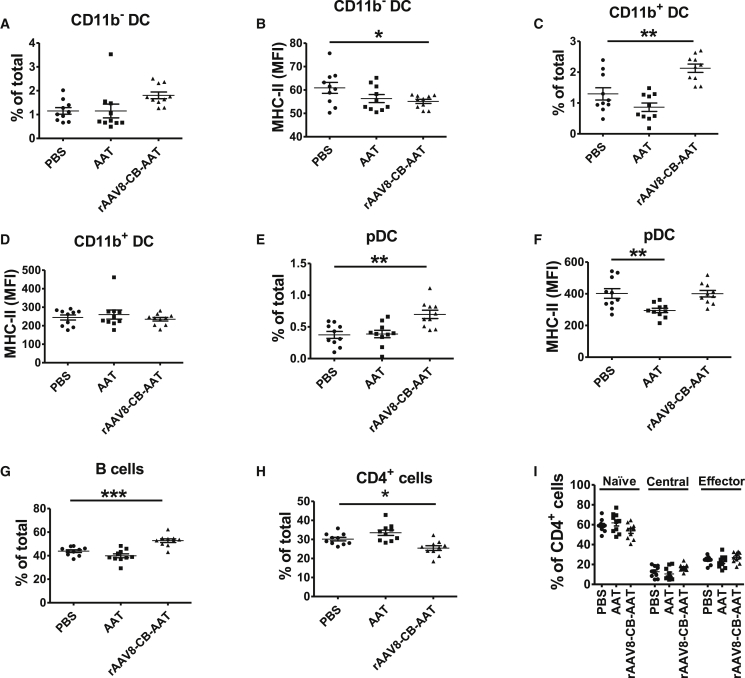
Effect of hAAT Treatment on NZM2410 Splenocytes after 14 Weeks of Treatment Mice described in Figure 4 were sacrificed at 22 weeks. Splenic conventional DCs were identified as CD11c^+^ with CD11c^+^CD11b^−^ (presented as CD11b^−^) and CD11c^+^CD11b^+^ (presented as CD11b^+^) subsets, plasmacytoid DCs (pDC) as PDCA-1^+^CD11c^int^, and B cells as CD19^+^ cells. The frequency of these cells was calculated relative to total live splenocytes. CD4^+^ T cells were subdivided into naive (CD62L^+^CD44^−^), central memory (CD62L^+^CD44^+^), and effector memory (CD62L^−^CD44^+^). (A) Frequency of CD11b^−^ DCs. (B) Expression of MHC-II on CD11b^−^ DCs (gated on CD11c^+^CD11b^−^ DCs). PBS versus rAAV-CB-hAAT was analyzed by two-tailed unpaired t test. (C) Frequency of CD11b^+^ DCs. (D) MHC-II expression on CD11b^+^ DCs indicated as MFI (gated on CD11c^+^CD11b^+^ DCs). (E) Frequency of pDC (PDCA-1^+^CD11c^int^, gated on total live cells). (F) MHC-II MFI expression on pDCs (gated on pDCs). (G) Frequency of B cells (CD19^+^ cells gated on total live cells). (H) Frequency of CD4^+^ cells (CD4^+^ cell gated on total live cells). (I) Frequency of naive (CD62L^+^CD44^−^), central memory (CD62L^+^CD44^+^), and effector memory (CD62L^−^CD44^+^) T cells (gated on CD4^+^ T cells). Data were expressed as the mean ± SEM for ten mice per group and analyzed by one-way ANOVA using Tukey’s post hoc test. *p < 0.05, **p < 0.01, ***p < 0.001.

We next characterized cDC subsets (CD11c^+^CD11b^−^ presented as CD11b^−^ and CD11c^+^CD11b^+^ presented as CD11b^+^), pDCs (PDCA-1^+^CD11c^int^), and B (CD19^+^) and CD4^+^ T cells in the spleen of AAT-treated and control mice. AAT protein or gene therapy did not affect the frequency of CD11b^−^ cells (Figure 5A), but rAAV8-CB-hAAT treatment reduced the expression of the activation marker MHC-II on CD11b^−^ DCs (Figure 5B). Interestingly, rAAV8-CB-hAAT treatment increased the frequency of CD11b^+^ cDCs and pDCs (Figures 5C and 5E), while it did not affect the expression of MHC-II on CD11b^+^ cDCs or pDCs (Figures 5D and 5F). However, hAAT protein therapy significantly reduced the expression of the MHC-II activation marker on pDCs (Figure 5F). hAAT protein therapy did not affect the frequency of B and CD4^+^ T cells (Figures 5G and 5H). hAAT gene therapy increased the percentage of CD19^+^ B cells in the spleen (Figure 5G), while it did not affect the frequency of class-switched B cells or germinal center class-switched B cells (data not shown), which are B cell subsets that are expanded in lupus and normally associated with anti-dsDNA IgG production. hAAT gene therapy significantly reduced the frequency of CD4^+^ T cells (Figure 5H). Neither hAAT protein nor gene therapy showed a significant effect on the distribution of naive (CD62L^+^CD44^−^), central memory (CD62L^+^CD44^+^), or effector memory (CD62L^−^CD44^+^) cells CD4^+^ T cells (Figure 5I). Overall, these results indicate that hAAT protein or gene therapy has a mild effect at this early time point (22 weeks of age).

### hAAT Gene Therapy Prevented Lupus Development and Extended the Lifespan of NZM2410 Mice

**Figure 6:**
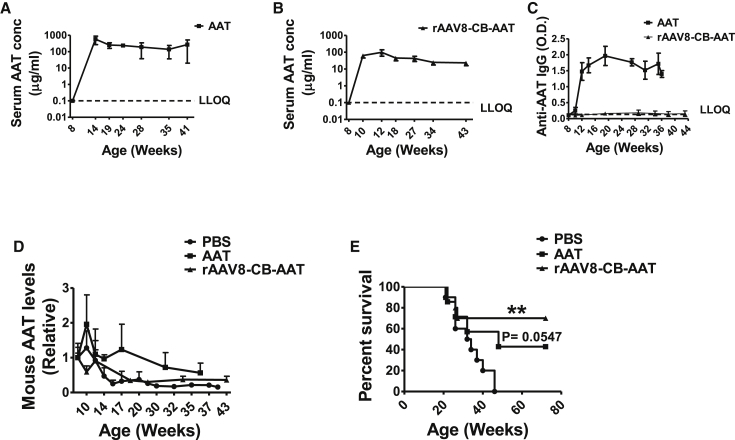
hAAT Gene Therapy Extends the Lifespan of NZM2410 Mice 8-week-old female NZM2410 mice were injected with PBS (n = 10) as a control, hAAT (n = 7, 2 mg/mouse, every 3 days), and rAAV8-CB-hAAT (n = 10, 1 × 10^11^ particles/mouse). The experiment was terminated at 72 weeks of age. (A) Serum hAAT levels in the hAAT-treated group. (B) Serum hAAT levels in the rAAV8-CB-hAAT-treated group. (C) Anti-hAATIgG levels. (D) Relative murine AAT levels (relative unit to C57BL/6 levels) in PBS-, hAAT-, and rAAV8-CB-hAAT-treated NZM2410 mice. (E) Kaplan-Meier curves showing survival of mice treated with PBS alone or hAAT or rAAV8-CB-hAAT. Data were expressed as the mean ± SEM. p (PBS versus hAAT) = 0.0547 and **p (PBS versus rAAV8-CB-hAAT) < 0.01.

To further elucidate the long-term effect of hAAT, we performed a survival experiment. Female NZM2410 mice were treated starting at 8 weeks old with hAAT as protein therapy, rAAV8-CB-hAAT vector as a gene therapy, or PBS as a control. An animal was sacrificed when it developed severe disease, defined as showing two successive >300 mg/dL proteinuria or losing more than 15% of body weight within a week. The experiment was terminated at 72 weeks of age. As in the short-term study, we monitored serum levels of hAAT in the protein therapy group (∼200 μg/mL; Figure 6A) and in the gene therapy group (50–100 μg/mL; Figure 6B). We also monitored anti-hAAT antibodies (Figure 6C).

Consistent with previous observation, anti-hAAT IgG was not detectable in the rAAV8-CB-hAAT-treated group. Interestingly, serum murine AAT levels decreased gradually in all groups as the disease developed, indicating the insufficiency of endogenous mouse AAT and the necessity of exogenous AAT (e.g., hAAT treatment) in this disease condition (Figure 6D). Similar to previous reports,^37^, ^38^ NZM2410 mice in the control group started to develop severe lupus at 21 weeks of age, and all the animals died by 46 weeks of age (Figure 6E). In contrast, 42% of hAAT-treated mice and 70% of rAAV8-CB-hAAT-treated mice remained healthy until 72 weeks of age (Figure 6E). These results clearly showed that hAAT treatment, especially hAAT gene therapy, protected against lupus development and extended the lifespan of lupus-prone mice.

## Discussion

SLE is a complex autoimmune disease in which DCs play critical roles in the disease pathogenesis.^7^, ^39^ Therefore, targeting DC activation and function could have therapeutic potential in the treatment of SLE. Standard treatments involve non-steroidal anti-inflammatory drugs (NSAIDs) and antimalarial and immunosuppressive agents, which are nonspecific and have severe side effects.^40^ There is an unmet need to develop effective therapy with no or minimal side effects. hAAT is a multifunctional protein that plays a vital role in modulating the immune response and controlling inflammation.^19^, ^21^, ^23^, ^41^, ^42^ hAAT is FDA approved and currently used to treat alpha 1 antitrypsin-deficient (AATD) patients.^42^ We have shown that hAAT treatment inhibited the activation and functions on non-autoimmune BMDCs and reduced lupus development in the MRL/lpr mouse model.^25^ In this study, we showed that hAAT treatment inhibited the activation and function of BMDCs derived from another lupus-prone mouse, B6.TC. We also showed that the indirect effect of hAAT-treated DCs on B cell differentiation and antibody production was greater than the direct effect of hAAT on B cells. Most importantly, we showed that hAAT protein therapy or gene therapy (rAAV8-CB-hAAT) decreased the production of autoantibodies and can extend the lifespan of lupus-prone mice. These promising results indicate the therapeutic potential of hAAT for the treatment of SLE in humans.

DCs play an important role in the pathogenesis of lupus. DCs from SLE patients showed an altered expression of costimulatory molecules CD40 and CD86 as compared with healthy controls.^43^, ^44^ Moreover, IL-12 produced by DCs induces the differentiation of Th1 cells, which are expanded in autoimmune diseases,^45^ and targeting IL-12 modulates autoimmune diseases in animal models.^46^, ^47^ Using the B6.TC lupus-prone mouse model, we investigated the effect of hAAT treatment on BM-derived cDCs and pDCs *in vitro*. Consistent with our previous observations in DCs from non-autoimmune mice, we showed that hAAT treatment inhibited the expression of MHC-II molecules, critical for antigen presentation and processing, as well as costimulatory molecules (CD80, CD86, and CD40). CCR9 is expressed by a tolerogenic subset of pDCs, which induced regulatory T cells and inhibited antigen-specific immune responses.^35^ In this study, we showed that hAAT treatment significantly upregulated the frequency of the CCR9+ pDC population. Consistent with previous observations,^25^, ^48^ these results indicate that the hAAT treatment induces tolerogenic DCs. hAAT treatment also reduced the production of IFN-I, TNF-α, and IL-6, which are cytokines that have been associated with lupus.^7^, ^8^, ^49^ In addition, we showed for the first time that hAAT inhibited IL-12 production from DCs. These results not only provide more support to the notion that hAAT has a therapeutic potential but also shed light on understanding the mechanism of hAAT action. Since IL-12 is one of the early responsive cytokines from DCs,^50^ further investigations on how hAAT regulates IL-12 production in DCs will provide important insight into hAAT functions and lupus pathogenesis.

DCs have a critical effect on B cell development and function.^7^, ^51^ DCs produce BAFF, as well as cytokines such as IL-12, IL-6, and IFN-I that play an important role in B cell development.^12^ Moreover, activated DCs from B6.TC mice increase B cell function.^7^ It was suggested that DCs could act as a base for the activation and the interaction between B and T cells, leading to the production of antibodies.^52^ Here we showed that hAAT treatment inhibited B cell proliferation upon stimulation with TLR4 or TLR9 agonists but had minimal effect on total IgM antibody production. Moreover, we found that DCs co-cultured with B cells enhanced cell proliferation and IL-6 and B cell antibody secretion upon stimulation with TLR4, TLR7, or TLR9 agonists, consistent with a previous report.^7^ Importantly, using a co-culture system, we demonstrated that hAAT-treated DCs significantly inhibited B cell proliferation, total IgM production, and IL-6 production. These data indicated that hAAT may affect B cell function through its action on DCs.

SLE is a heterogeneous autoimmune disease, which is characterized by hallmark autoantibody production causing tissues damage.^53^ Therefore, reducing autoantibody production is one of the major therapeutic goals for the treatment of lupus. Besides currently used immunosuppressive therapies, which may lead to side effects, safe therapeutic options that can reduce autoantibodies are limited. We have previously shown that hAAT protein and gene therapy decrease autoantibody levels in mouse models of type 1 diabetes (NOD),^22^ arthritis (collagen-induced arthritis [CIA]),^21^ and lupus (MRL/lpr).^25^ Consistent with these observations, in this study we showed that treatments with both hAAT protein and gene therapy significantly reduced autoantibody levels (anti-dsDNA IgG) in NZM2410 mice. The minimal effective levels of hAAT required for the prevention or treatment of lupus also require further investigation. However, our results showed that the addition of 50–100 μg/mL hAAT is effective, consistent with what we observed in rheumatoid arthritis and osteoporosis mouse models.^19^, ^21^ It is possible that the disease leads to endogenous AAT insufficiency, including insufficient gene expression and loss of AAT functions, which is compensated by exogenous hAAT treatment. It should be noted that the effective levels in lupus are much lower than the therapeutic levels (572 μg/mL, 11 μM) for AATD patients, and they may be achievable in humans considering that 20 μg/mL has been achieved in human muscle with rAAV1-CB-hAAT.^54^ Importantly, no side effect was observed in all our studies, consistent with the safety profile from hAAT augmentation therapy and gene therapy in human clinical studies.^55^, ^56^ While the mechanism underlying the safety of hAAT requires further investigation, one possible explanation is that the exogenous hAAT levels resulting from gene therapy or augmentation therapy are in the physiological range, since AAT levels are normally high (1–3 mg/mL in the circulation) and can increase several fold in certain conditions, such as inflammation.

Compared with hAAT protein therapy, rAAV8-mediated AAT gene therapy showed several advantages. (1) While hAAT protein injection induced anti-hAAT antibody production as expected, rAAV8-CB-hAAT treatment resulted in undetectable levels of anti-hAAT antibody, consistent with our previous observations in NOD, CIA, and bone loss mouse models.^19^, ^21^, ^31^ Since foreign protein-induced immune response can alter the immune system and lead to nonspecific therapeutic effect, as well as limits its long-term efficacy, our results with hAAT gene therapy are critical to demonstrate the therapeutic effect of hAAT on lupus development in the NZM2410 mouse model. (2) Although serum hAAT levels in the rAAV8-CB-hAAT group is relatively lower than that in the hAAT protein-injected group, rAAV8-CB-hAAT treatment showed more pronounced immunoregulatory effects, including more reduction of anti-dsDNA, total IgM, and total IgG levels, as well as urine albumin, IL-12, and IFN-γ levels. While further investigations remain to be performed, several factors may contribute to this advantage. It is possible that a sustained level of hAAT from rAAV8 is more effective than the fluctuating levels of hAAT from protein injection. In addition, hAAT from rAAV8-transduced cells may be more protective locally in related tissues and organs. (3) Most importantly, rAAV8-CB-hAAT treatment resulted in a more significant therapeutic effect and extended lifespan up to 72 weeks. These results clearly demonstrated that long-term control of autoimmunity can be achieved by maintaining a sustained serum level of hAAT with hAAT gene therapy and the combination of its protective effects (anti-proteinase, anti-inflammatory, and immunoregulatory) may contribute to extending the lifespan. These advantages together with other common advantages of gene therapy (e.g., single-vector administration versus repeated protein injections, avoiding protein purification, and overcoming a shortage of protein resources) strongly indicated that rAAV8-mediated hAAT gene therapy has therapeutic potential for the treatment of autoimmune diseases in humans.

It has been reported that rAAV may activate TLR9 signaling.^57^ rAAV2 vector activates the TLR-MyD88 pathway in mouse pDCs,^58^ and TLR9 activation and depletion of regulatory T cells (Tregs) resulted in immune responses to the transgene product from rAAV1 vector in muscle.^59^ The presence of CpG nucleotides in the rAAV vector is critical for the recognition of TLR9 after intramuscular injection.^60^ However, it is important to note that the vector serotype makes major differences for the TLR9 response. While the transgene expression from AAVrh32 was completely blocked, transgene expression from rAAV8 was minimally affected in wild-type mice compared with that in TLR9^−/−^ mice.^60^ In line with the literature, our data showed that rAAV8-CB-AAT injection resulted in undetectable levels of anti-hAAT antibodies (Figure 4C), suggesting that rAAV8 vector may have a minimal effect, if any, on activating TLR9 in NZM2410 mice. Since TLR9 activation may enhance lupus disease development^61^ and make it harder to prevent it, our results showing a significant protective effect of rAAV8-CB-hAAT treatment indicate that TLR9 activation by rAAV8 vector in NZM2410 mice is negligible or counterbalanced by hAAT, which inhibits TLR9 signaling.^25^ Nonetheless, the effect of rAAV8-CB-hAAT vector DNA on TLR9 activation should be further investigated in additional lupus models before translating these results into clinical applications.^62^

In summary, we have shown that hAAT can inhibit DC activation and function, including IL-12 production, in lupus mouse models. The effects of hAAT on B cells are primarily through its action on DCs. Treatment with hAAT can prevent lupus development and extend the lifespan in NZM2410 mice. Finally, rAAV8-mediated hAAT gene therapy has significant advantages for the treatment of lupus.

## Materials and Methods

### Mice

The B6.NZM^*Sle1/2/3*^ (B6.TC) congenic strain has been previously described.^32^ Female NZM2410 and C57BL/6 mice were purchased from The Jackson Laboratory (Bar Harbor, ME). All mice were housed in specific pathogen-free (SPF) conditions and monitored daily. For experiments using BMDCs and B cells, we used 2- to 3-month-old mice. For both short-term and long-term studies, 8-week-old female NZM2410 mice were randomly divided into PBS, hAAT protein therapy (hAAT), or hAAT gene therapy (rAAV8-CB-hAAT) groups. In the PBS group, mice were intradermally (i.d.) injected with PBS (100 μL every 3 days, Corning Cellgro, Manassas, VA). In the AAT protein treatment group, mice were i.d. injected with hAAT (Prolastin C, Grifols, NC; 2 mg/mouse in 100 μL PBS, every 3 days). For the hAAT gene therapy group, mice were intraperitoneally (i.p.) injected with rAAV8-CB-hAAT vectors (single injection of 1 × 10^11^ particles/mouse in 100 μL Ringer’s lactated solution). Mice were sacrificed when they developed severe disease (i.e., showed two successive >300 mg/dL proteinuria), lost more than 15% of body weight within a week, or after a set endpoint (22 weeks old in the short-term study and 72 weeks old in the long-term study). All experiments were conducted according to protocols approved by the University of Florida Institutional Animal Care and Use Committee.

### cDC Preparation and Cell Cultures

BM cells from B6.TC mice were cultured with or without 1 mg/mL hAAT in complete RPMI 1640 (Corning Cellgro, Manassas, VA) containing 10% fetal bovine serum (FBS) (Thermo Scientific, USA), 2- Mercaptoethanol (Sigma-Aldrich, MO), 10 ng/mL GM-CSF, and 5 ng/mL IL-4 (PeproTech, NJ). On day 3, 50% of the culture medium was replaced with fresh medium containing the same supplements. On day 4, cells were stimulated by adding 10 μg/mL CpG-ODN 1826 (InvivoGen, San Diego, CA) for an additional 24 hr. All cells were collected at day 5 and analyzed by flow cytometry, and the supernatant was stored at −80°C for cytokine detection.

### pDC Induction

BM cells from B6.TC mice were plated at 2 × 10^6^ cells/well with or without 1 mg/mL AAT in RPMI 1640 medium supplemented with 10% heat-inactivated fetal bovine serum, 100 U/mL penicillin and streptomycin, 2-Mercaptoethanol, and 200 ng/mL Flt3L (R&D Systems, Minneapolis, MN). At day 4, half of the medium was replaced with fresh medium containing the same supplements. On day 8, cells were stimulated with or without CpG-ODN 1826 (InvivoGen, San Diego, CA) for an additional 24 hr. Then, the cells were harvested and analyzed by flow cytometry, and the supernatants were kept at −80°C for cytokine detection.

### B Cell Isolation

B cells were purified from the spleen of C57BL/6 mice by CD43-negative selection (Miltenyi Biotec, Auburn, CA). Purity of the CD19^+^ B220^+^ fraction was >90%. CD11c+ cells were purified from C57BL/6 BM cDCs by CD11c-positive selection (Miltenyi Biotec, Auburn, CA). The purity of CD11c^+^ MHC-II I-A^b +^ was >97%.

### DC and B Cell Co-cultures

Splenic B cells (10^5^) from C57BL/6 mice were co-cultured with purified CD11c^+^ cells from BMDCs (2 × 10^4^) from C57BL/6, treated with or without hAAT. The co-culture was performed for 5 days in 96-well plates (Corning, NY) in complete RPMI, in the presence or absence of 0.5 μg/mL LPS (Sigma-Aldrich, MO), 5 μg/mL Imiquimod (InvivoGen, San Diego, CA), or 10 μg/mL CpG-ODN1826 (InvivoGen, San Diego, CA). After 5 days, 100 μL supernatant was collected and kept at –80°C for cytokine and antibody detection and cell proliferation assessment. Cell proliferation was assessed by CellTiter 96 AQ_ueous_ One Solution Cell Proliferation Assay (Promega, Madison, WI).

### Flow Cytometry

BM cDCs and BM pDCs from B6.TC or splenocytes from NZM2410 mice were blocked with Fc receptor blocker, anti-CD16/CD32, (2.4 G.2) to block FcR-mediated binding. Cells were then stained with monoclonal antibodies to mouse CCR9 (CW-1.2), CD11b (M1/70), CD11c (HL3), CD40 (3/23), CD80 (16/10 A1), CD86 (GL1), PDCA-1 (927), MHC-II (AF6-120.1), CD19 (1D3-CD19), CD4 (RM4-5), I-A^b^ (M5/144.15.2), CD44 (IM7), CD62L (AF6-78), IgM^b^ (MEL-14), B220 (RA3-6B2), and GL-7 (GL7). All antibodies were obtained from BD Biosciences (San Diego, CA), BioLegend (San Diego, CA), or eBioscience (San Diego, CA). Biotin-conjugated antibodies (Abs) were revealed using streptavidin-PERCP-Cy 5.5 (BD Biosciences, San Diego, CA). At least 100,000 cells were acquired and analyzed using a FACSCalibur (BD Biosciences). Flow cytometry datasets were analyzed with the FCS Express software (De Novo), and dead cells were excluded by forward and side scatter characteristics.

### Cytokine Assays

IL-6, TNF-α, and IL-12 in cell culture medium were detected by ELISA (PeproTech, NJ). IFN-I was quantified using murine IFN-I sensor B16-Blue IFN-α/β cells (InvivoGen, San Diego, CA), as described previously.^25^ Briefly, 20 μL culture media was combined with 180 μL medium containing B16-Blue IFN α/β cells (7.5 × 10^4^) in each well of a 96-well plate. The plate was incubated at 37°C in 5% CO_2_ for 22–24 hr. On the second day, 180 μL QUANTI-Blue was combined with 20 μL induced B16-Blue IFN α/β cell supernatant in a flat-bottom 96-well plate, which was incubated at 37°C for 3–4 hr. The levels of purple and blue color were detected at 630 nm.

### Detection of Serum hAAT Concentration and Anti-hAAT Antibody

Serum hAAT levels were detected by hAAT-specific ELISA as previously described.^63^ Anti-hAAT antibodies were detected by an ELISA as previously described.^63^ Blood samples were collected weekly at 1 day after hAAT injection, except for the terminal sample collected 3 days after hAAT injection.

### Detection of Mouse AAT by ELISA

Mouse AAT levels in NZM2410 lupus mouse serum were detected by ELISA as previously described.^25^ Briefly, pooled B6 (adult male) mouse serum was used as a standard, in which mouse AAT concentration was defined as one relative unit. Samples (sera from lupus mice) and standards were diluted and incubated in a microtiter plate (Immulon 4, Dynex Technologies) in Voller’s buffer overnight at 4°C. The plate was blocked with 3% BSA (Sigma) for 1 hr at 37°C. Goat anti-mouse AAT antibody (1:2,000 dilution, MyBiosource, San Diego, CA) was added and incubated for 1 hr at 37°C. Then, horseradish peroxidase (HRP)-conjugated anti-goat-IgG antibody (1:2,500 dilution, R&D Systems, Minneapolis, MN) was added and incubated for 1 hr at 37°C. The plate was washed with PBS-Tween 20 between reactions. After adding substrate (O-Phenyldiamine, Sigma-Aldrich, MO), the plate was read at 490 nm on an MRX microplate reader (Dynex Technologies). The optical density (OD) reading of each sample was used to calculate relative unit based on the standard curve.

### Evaluation of Glomerulonephritis

Mice were evaluated for proteinuria using Albustix strips (Siemens, Tarrytown, NY). Urine samples were graded using a 0–4 scale (0, negative; 1, 30 mg/dL; 2, 100 mg/dL; 3, 300 mg/dL; and 4, over 2,000 mg/dL urinary protein). Mice with a significant proteinuria (>300 mg/dL) on 2 consecutive weeks were considered positive for renal disease. Severely ill mice with a significant proteinuria were sacrificed. These mice were included in the survival evaluation and considered as dead at the time they were sacrificed. Urinary albumin excretion was determined by ELISA (Kamiya Biomedical). Kidneys were fixed in 10% buffered formalin, embedded in paraffin, and sections were stained with Periodic acid-Schiff (PAS). The average of area and number of glomeruli were measured with image analysis software (Aperio Imagescope). Evaluation was performed by a pathologist who was blinded to the diagnosis.

### Detection of Anti-dsDNA, Total IgG, and Total IgM Antibodies

Anti-dsDNA IgG was detected as previously described.^25^, ^32^ Relative units were standardized to a 1:100 dilution of a B6.TC serum set to 100 units and run in serial dilution on each plate. Total IgG was detected in NZM2410 serum by sandwich ELISA as described before.^32^ Briefly, Immulon 2 B plates (Thermo Scientific) were pre-coated with goat-anti mouse IgG (Millipore, MA) at 1 μg/mL in 0.1 M bicarbonate buffer, washed, and blocked with 0.1% gelatin containing 3% BSA/3 mM EDTA. Diluted serum was added (1:1,000 in 0.1% gelatin containing 2% BSA, 3 mM EDTA, and 0.05% Tween 20). Goat anti-mouse IgG-alkaline phosphatase (AP) (Millipore, MA) was used as a secondary antibody at a 1:1,000 dilution. AP activity was detected by using para-nitrophenyl phosphate (pNPP) substrate in 0.025 M bicarbonate buffer with 1 M MgCl_2_.

Total IgM was detected by sandwich ELISA as described previously.^11^ Immulon 1 plates (Thermo Scientific) were pre-coated with goat anti-mouse IgM (Millipore, MA) at 1 μg/mL in 0.1 M bicarbonate buffer, washed, and blocked with 0.1% gelatin containing 3% BSA/3 mM EDTA. Mouse serum or culture supernatant samples were diluted 1:1,000 or 1:10, respectively, in 0.1% gelatin containing 2% BSA, 3 mM EDTA, and 0.05% Tween 20. Goat anti-mouse IgM-AP (Southern Biotechnology, Birmingham, AL) was used as a secondary antibody at 1:5,000 dilution. AP activity was detected as before using pNPP substrate.

### Statistical Analysis

Data analysis was performed with GraphPad Prism software package version (v.)5.04 (La Jolla, CA, USA) with the tests indicated in the text. Data were subjected to ANOVA with Tukey’s post hoc test, two-tailed Student’s t test, or Mann-Whitney test or as indicated in the text. Graphs show mean and SEM, and significance levels in figures are presented as *p < 0.05, **p < 0.01, and ***p < 0.001.

## Author Contributions

Study Design, A.S.E., L.M., and S.S.; Methodology, A.S.E., G.A., Y.Y., Y.L., H.P., T.H., H.W., L.Z., and E.T.; Data Analysis and Interpretation, A.S.E., G.A., M.A.A., M.-J.C., L.Z., L.M., and S.S.; Manuscript Preparation, A.S.E., G.A., M.-J.C., L.M., and S.S.; Statistical Analysis, A.S.E., Y.Y., M.-J.C., L.M., and S.S.; Funding, L.M. and S.S.

## Conflicts of Interest

The authors have no conflicts of interest.
